# A rational two-step approach to *KRAS* mutation testing in colorectal cancer using high resolution melting analysis and pyrosequencing

**DOI:** 10.1186/s12885-016-2589-2

**Published:** 2016-08-02

**Authors:** Elisabeth Mack, Kathleen Stabla, Jorge Riera-Knorrenschild, Roland Moll, Andreas Neubauer, Cornelia Brendel

**Affiliations:** 1 Klinik für Hämatologie, Onkologie und Immunologie, Universitätsklinikum Gießen und Marburg, Standort Marburg, Philipps-Universität Marburg, Baldingerstraße, Marburg, Germany; 2 Institut für Pathologie, Universitätsklinikum Gießen und Marburg, Standort Marburg, Philipps-Universität Marburg, Baldingerstraße, Marburg, Germany

**Keywords:** *KRAS* mutation, Colorectal cancer, High resolution melting analysis, Pyrosequencing

## Abstract

**Background:**

*KRAS* mutation testing is mandatory in the management of metastatic colorectal cancer prior to treatment with anti-EGFR antibodies as patients whose tumors express mutant *KRAS* do not benefit from these agents. Although the U.S. Food and Drug Administration has recently approved two *in-vitro* diagnostics kits for determination of *KRAS* status, there is generally no consensus on the preferred method and new tests are continuously being developed. Most of these techniques focus on the hotspot mutations at codons 12 and 13 of the *KRAS* gene.

**Methods:**

We describe a two-step approach to *KRAS* codon 12/13 mutation testing involving high resolution melting analysis (HRM) followed by pyrosequencing using the *Therascreen KRAS* Pyro kit (Qiagen) of only those samples that are not clearly identified as *KRAS* wildtype or mutant by HRM. First, we determined *KRAS* status in a panel of 61 colorectal cancer samples using both methods to compare technical performance and concordance of results. Subsequently, we evaluated practicability and costs of our concept in an independent set of 120 colorectal cancer samples in a routine diagnostic setting.

**Results:**

HRM and pyrosequencing appeared to be equally sensitive, allowing for clear detection of mutant alleles at a mutant allele frequency ≥12.5 %. Pyrosequencing yielded more exploitable results due to lower input requirements and a lower rate of analysis failures. *KRAS* codon 12/13 status was called concordantly for 98.2 % (56/57) of all samples that could be successfully analysed by both methods and 100 % (19/19) of samples that were identified mutant by HRM. Reviewing the actual effort and expenses for *KRAS* mutation testing in our laboratory revealed, that the selective use of pyrosequencing for only those samples that could not be analysed by HRM increased the fraction of valid results from 87.5 % for HRM alone to 99.2 % (119/120) while allowing for a net reduction of operational costs of >75 % compared to pyrosequencing alone.

**Conclusions:**

Combination of HRM and pyrosequencing in a two-step diagnostic procedure constitutes a reliable and economic analysis platform for *KRAS* mutation testing in colorectal cancer in a clinical setting.

**Electronic supplementary material:**

The online version of this article (doi:10.1186/s12885-016-2589-2) contains supplementary material, which is available to authorized users.

## Background

The anti EGFR-antibodies cetuximab and panitumumab represent well-established treatments for metastatic colorectal cancer (CRC), the third most prevalent cancer entity and fourth most common cause of cancer-related death around the world [1, 2]. Several studies have shown *KRAS* status to predict outcome under these anti-EGFR targeting agents, with beneficial effects being seen only in patients whose tumors express wildtype (WT) *KRAS* [3–8]. Thus, testing for *KRAS* mutations, which are found in approximately 40 % of colorectal cancers, has become routine in the management of metastatic CRC (mCRC) prior to cetuximab or panitumumab treatment [9, 10] and is even required by the responsible regulatory agencies. Notably, current standards regarding oncogenic Ras mutation analysis in mCRC issued by the U.S Food and Drug Administration (FDA) require determination of *KRAS* status by an FDA-approved test, while the European Medical Agency (EMA) just states application of validated methods by an experienced laboratory [11–15]. Currently available FDA-approved companion diagnostic devices for cetuximab (Erbitux) and panitumumab (Vectibix) comprise the Cobas *KRAS* Mutation Test (Roche) and *Therascreen KRAS* RGQ PCR Kit (Qiagen) [16]. Besides these and other commercially available kits, the spectrum of methods for *KRAS* mutation testing encompasses multiple PCR-derived and sequencing-based techniques. Of note, most of the previously established assays for *KRAS* mutation detection focus on the hotspot mutations involving codons 12 and 13, which account for >95 % of Ras mutations in CRC [10]. The advantages and limitations of selected methods have been repeatedly evaluated comparatively [17–22], however, beyond the FDA-guideline, there is no consensus on the preferred approach to investigate *KRAS* status in routine molecular pathological diagnostics [23]. Given the high incidence of CRC resulting in high demand for *KRAS* mutation testing, an ideal diagnostic assay for this purpose not only needs to be sufficiently sensitive and specific, but, for socio-economic reasons, also should be time- and cost-effective. Therefore, we developed a two-step procedure for *KRAS* mutation testing including high resolution melting analysis (HRM) followed by pyrosequencing of only those samples that are not clearly identified as *KRAS* WT or mutant by HRM. HRM is a one-tube qPCR-based technique for DNA-variant detection. The method utilizes alterations in the melting behavior of double-stranded DNA fragments that are conferred by nucleotide exchanges. Melting of qPCR amplicons is monitored in real time using a suitable qPCR instrument capable of time-dense data aquisition and a saturating DNA-intercalating fluorescent dye that does not redistribute during the melting step [24]. Pyrosequencing is a sequencing-by-synthesis approach that involves sequential addition of dNTPs and recording incorporation of a nucleotide based on a light signal that is generated by sulfurylase-catalyzed conversion of the released pyrophosphate to ATP and a subsequent luciferase reaction [25]. Here, we applied a previously described HRM-assay [20] and the *Therascreen KRAS* Pyro kit (Qiagen) for detection of *KRAS* codon 12/13 mutations. First we comparatively analysed *KRAS* status in a panel of 61 colon cancer samples to determine sensitivity, specificity, technical performance and concordance of results of the two methods. Subsequently, we evaluated our two-step approach in the routine setting of our molecular diagnostics laboratory. In summary, we present a reliable, time- and cost-effective operational concept for *KRAS* mutation testing prior to anti-EGFR antibody treatment in mCRC.

## Methods

### Tumor samples, control cell lines and DNA isolation

The colorectal cancer samples reported on in this study were obtained from patients with metastatic colorectal cancer (UICC IV) at the University Hospital Marburg, Germany and analysed in a routine diagnostic setting. Tissue samples were fixed, paraffin-embedded, sectioned, hematoxylin-eosin stained and deparaffinated using standard procedures. Tissue sections were reviewed by an experienced pathologist (RM) to establish the diagnosis and to mark regions for microdissections. Microdissection of tumor cells was performed from deparaffinated sections using a scalpel. DNA was isolated from microdissected samples using the QiaAmp DNA Mini kit (Qiagen) as recommended by the manufacturer. *KRAS* mutant cell lines PL45 (pancreatic adenocarcinoma) and RPMI 8226 (multiple myeloma) were obtained from ATCC and cultured according to standard cell culture methods. Positive control DNA for HRM analyses was isolated from these cell lines using the QiaAmp DNA Mini kit. WT control DNA was extracted from peripheral blood of healthy donors from whom informed consent had been obtained (WT control) with the QiaAmp DNA Mini kit. DNA concentrations were measured using a Nanodrop 1000 spectrophotometer (Peqlab).

### High resolution melting analysis

For HRM analysis, a 92 bp amplicon spanning exons 2 and 3 of the *KRAS* gene was amplified from 60 ng (or less) of sample DNA using the primers *KRAS*-92_F 5′-ttataaggcctgctgaaaatgactgaa-3′ and *KRAS*-92_R 5′-tgaattagctgtatcgtcaaggcact-3′ [20], the DNA-intercalating dye SYTO 9 (Thermo) in a final concentration of 5 μM and Platinum Taq polymerase (Thermo). Amplification and melting analysis was performed on a Rotor Gene 6000 instrument (Corbett Life Sciences) under the following temperature conditions: one cycle 95 °C/2 min, 40 cycles 95 °C/15 sec – 67.5 °C/15 sec - 72 °C/15 sec, one cycle 95 °C/1 sec, pre-melt conditioning at 72 °C/90 sec, HRM-ramp from 72 °C to 95 °C rising at 0.2 °C per step/wait 2 sec each step. Controls in each HRM run included a no-template-control, a WT control (gDNA from healthy donor) and two mutation controls (gDNA from the cell lines RPMI 8226, *KRAS* codon 12 GGT → GCT/heterozygous, corresponding to G12A and PL45, *KRAS* codon 12 GGT → GAT/heterozygous, corresponding to G12D). All HRM assays were performed in quadruplicate.

### Pyrosequencing

Pyrosequencing of the *KRAS* codon 12/13 region was performed using the *Therascreen KRAS* Pyro Kit (Qiagen) as recommended by the manufacturer. 2 ng of DNA were used per analysis. PCR amplification of the target region was performed on a T-100 thermocycler (Biorad). For the pyrosequencing reaction on the PyroMark Q24 platform (Qiagen), amplicons were immobilized to the wells of a PyroMark Q24 plate using streptavidin high performance beads (GE Healthcare). Pyrosequencing results were analysed using the PyroMark Q24 software version 2.0 with the *Therascreen* KRAS Pyro-plugin report, which already incorporated the thresholds for mutation calls (detection limit for the mutation (LOD) + 3 %).

### Statistical analysis

HRM and pyrosequencing results were compared by contingency table analysis test using GraphPad Prism 5 software (GraphPad). Technical performance (1st run success vs. 1st run failure) was evaluated by two-sided Fisher’s exact test at a significance level of 5 %. The agreement between HRM and pyrosequencing results was quantified by kappa using the appropriate Graphpad Prism online calculator (http://graphpad.com/quickcalcs/kappa2).

## Results

### Sensitivity of HRM and pyrosequencing

**Fig. 1:**
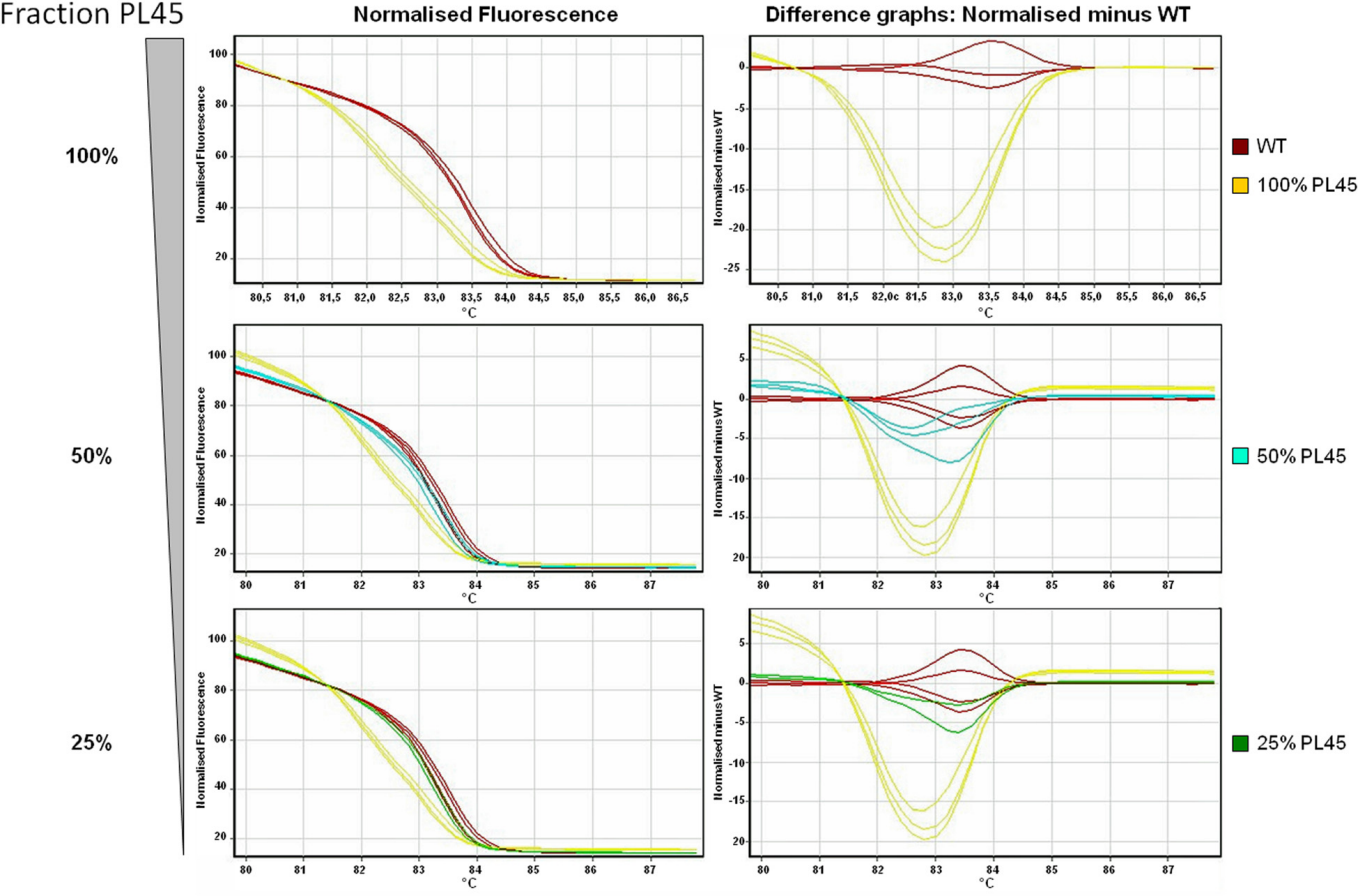
Sensitivity of HRM. Dilutions of genomic DNA from the *KRAS* mutant cell line PL45 (codon 12 GGT → GAT, heterozygous mutation) in WT genomic DNA was analysed by HRM. Normalised fluorescence and difference graphs are indicated. Clear discrimination of WT and mutant amplicons is possible using either graph if the fraction of PL45-DNA in the sample exceeds 25 %, corresponding to a mutant allele frequency of 12.5 %

**Fig. 2:**
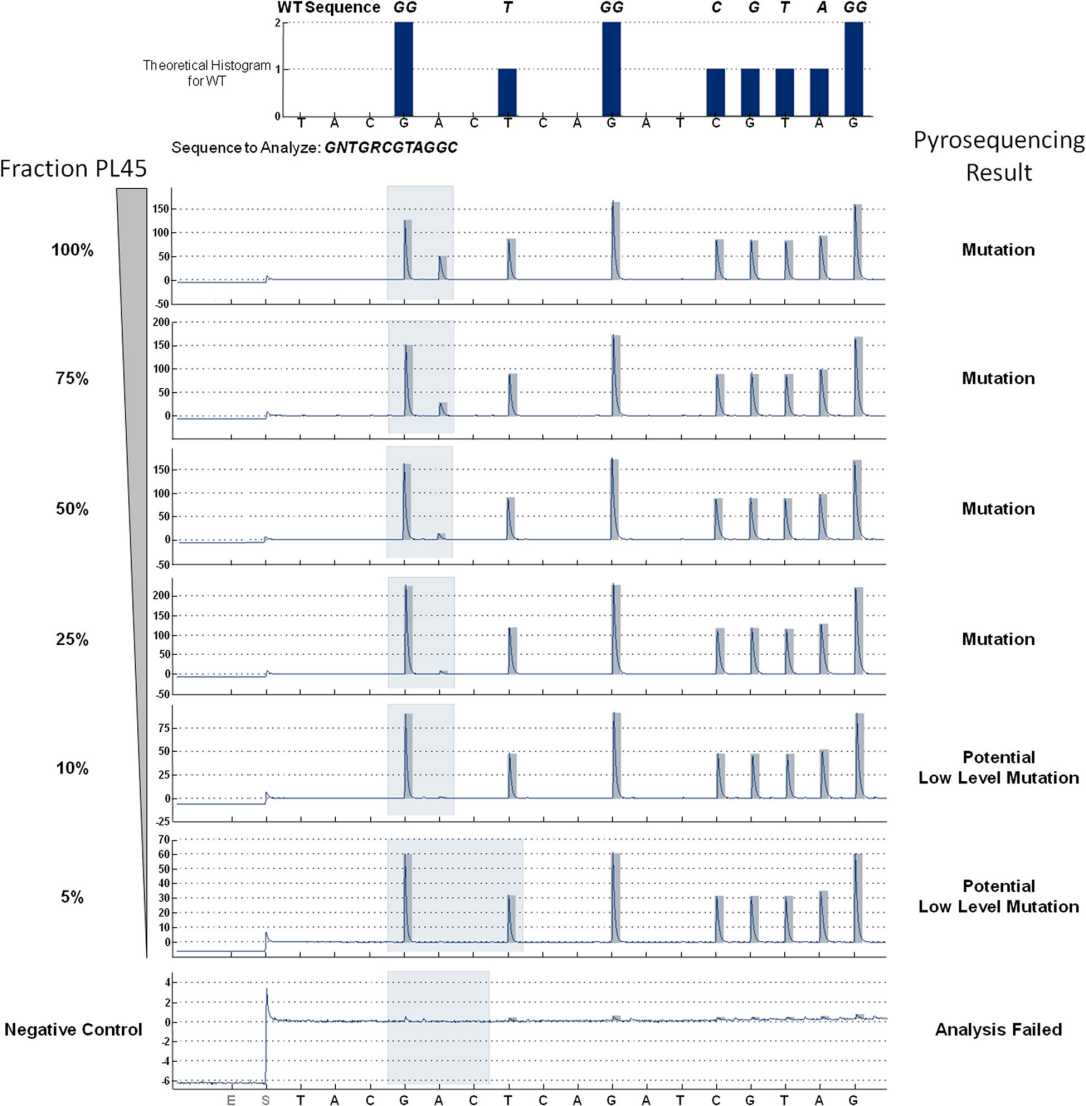
Sensitivity of pyrosequencing. Dilutions of genomic DNA from the *KRAS* mutant cell line PL45 (codon 12 GGT → GAT, heterozygous mutation) in WT genomic DNA was analysed by pyrosequencing on the Pyromark Q24 platform. The WT sequence and the sequence to analyse including wobble bases at the potentially mutant positions in the region of interest (codons 12 and 13) are indicated in the top panel. Lower panels: KRAS codon 12/13 pyrograms for different dilutions of PL45-DNA in WT DNA. Clear mutation calls are obtained for samples containing ≥25 % of PL45-DNA, corresponding to 12.5 % mutant alleles. For lower concentrations, yielding signals below the LOD of the mutation (2.2 %) + 3 %, presence of a potential low level mutation is suggested, which requires technical and/or biological replication of the analysis

In order to test whether pyrosequencing allows for *KRAS* mutation detection with at least equal sensitivity compared to HRM, we analysed serial dilutions of DNA from a *KRAS* mutant cell line (PL45, codon 12 GGT → GAT heterozygous mutation) in WT DNA by both HRM and pyrosequencing. For HRM, we found, that the presence of *KRAS* mutant DNA in the sample was clearly reflected by a shifted or skewed melting curve for a fraction of PL45-DNA exceeding 25 %, which corresponded to a mutant allele frequency of 12.5 % (Fig. 1). Similarly, pyrosequencing definitely yielded a mutation if the sample contained ≥25 % PL45-DNA. On the other hand, samples with 5–10 % cell line DNA were indicated to exhibit a potential low level mutation as the mutant allele frequency was quantified below the threshold for accurate WT/mutant discrimination for the G12D mutation (LOD + 3 %; LOD = 2,2 %) for both the 5 and 10 % samples (Fig. 2). Thus, HRM and pyrosequencing appeared to be equally sensitive methods for the detection of KRAS codon 12/13 mutations.

### Technical reliability of HRM and pyrosequencing

To further assess the suitability of pyrosequencing to serve as a backup-assay allowing for accurate diagnosis of *KRAS* mutation status in case of failed HRM analysis, we investigated *KRAS* status of 61 colorectal cancer samples by both HRM and pyrosequencing and compared the two methods with regard to their technical performance and concordance of results. In a first run of HRM analysis, 11/61 samples (18.0 %) could not be analysed due to PCR-failures or ambiguous melting curves (Table 1). Repetition of the assay for seven samples, which most likely had been compromised technically, allowed for assigning *KRAS* mutation status in all cases. The remaining four samples were directly subjected to pyrosequencing without a second round of HRM analysis. Indeed, *KRAS* status could each be determined, although one sample yielded a potential low level mutation. Of the 57 samples that could be definitely classified as *KRAS* WT or mutant by HRM, 26 WT samples (45.6 % of all samples/68.4 % of WT samples) yielded skewed HRM curves, which, however, did not prevent establishment of a diagnosis (Tables 1 and 2). Moreover, we noted that low DNA content of the samples below the detection limit of the Nanodrop spectrophotometer not necessarily prevented successful HRM analysis. In contrast to HRM, the pyrosequencing assay had to be repeated for only one sample (Table 1). Thus, the failure rate of a first analysis run as a consequence of technical and/or sample-issues was significantly higher for HRM analysis than for pyrosequencing (*p* = 0.0042). Together, pyrosequencing is technically more reliable than HRM due to lower input requirements and a lower incidence of invalid results.

**Table 1:** *KRAS* codon 12/13 status by HRM and pyrosequencing in 61 CRC samples

		HRM		Pyrosequencing				
				Run 1		Run 2		
Sample	cDNA [ng/μl]	Run 1	Run 2	Result	% mut. Alleles	Result	% mut. Alleles	Final result
1	11	failed	WT ^a^	WT				WT
2	31	WT ^a^		WT				WT
3	10	WT ^a^		G12C	13.4			mut
4	93	WT ^a^		WT				WT
5	26	WT ^a^		WT				WT
6	27	WT ^a^		WT				WT
7	10	WT ^a^		WT				WT
8	43	mut		G13D	73.9			mut
9	85	failed	WT	WT				WT
10	N/A	mut		G13D	44.3			mut
11	N/A	failed		G12V	2.6			WT ^b^
12	N/A	WT		WT				WT
13	N/A	mut		G12V	41.9			mut
14	126	mut		failed		G12D	65.7	mut
15	133	mut		G12D	74.1			mut
16	97	mut		G13D	52.8			mut
17	47	failed	WT	WT				WT
18	14	failed	WT	WT				WT
19	44	WT ^a^		WT				WT
20	20	failed	WT ^a^	WT				WT
21	N/A	failed		WT				WT
22	138	mut		G12V	12.9			mut
23	325	mut		G12D	56.1			mut
24	140	WT ^a^		WT				WT
25	7	mut		G12C	61.2			mut
26	4	WT ^a^		WT				WT
27	27	WT ^a^		WT				WT
28	13	WT ^a^		WT				WT
29	207	mut		G12D	54.5			mut
30	10	failed	WT	G12V	1.5			WT ^b^
31	54	WT ^a^		WT				WT
32	120	mut		G12V	29.2			mut
33	33	failed	mut	G12C	33.5			mut
34	N/A	mut		G12C	71.4			mut
35	N/A	WT		WT				WT
36	N/A	failed		WT				WT
37	67	WT ^a^		WT				WT
38	137	mut		G12C	76.7			mut
39	63	mut		G12A	56			mut
40	13	WT ^a^		WT				WT
41	113	WT ^a^		WT				WT
42	82	mut		G12C	75.4			mut
43	39	WT		WT				WT
44	7	WT ^a^		G12S	2			WT ^b^
45	23	WT ^a^		WT				WT
46	24	failed^a^		G13D	3.5			WT ^b^
47	9	WT ^a^		WT				WT
48	34	WT ^a^		G12S	2			WT ^b^
49	25	WT ^a^		WT				WT
50	17	WT		WT				WT
51	5	WT ^a^		WT				WT
52	29	WT ^a^		WT				WT
53	31	mut		G12D	71.3			mut
54	68	WT		WT				WT
55	93	mut		G12D	74.2			mut
56	221	WT		WT				WT
57	83	WT		WT				WT
58	N/A	WT ^a^		G12V	1.2			WT ^b^
59	35	mut		G12D	83.3			mut
60	N/A	WT		WT				WT
61	37	WT ^a^		WT				WT

^a^Skewed HRM curve

^b^LOD/threshold for potential low level mutation (cf. *Therascreen* KRAS Pyro Kit handbook version 1, July 2011): G12D 2.2 %/5.2 %, G12V 1.0 %/4.0 %, G12C 2.1 %/5.1 %, G12S 1.9 %/4.9 %, G13D 1.9 %/4.9 %

**Table 2:** Comparison of HRM and pyrosequencing results in 61 CRC samples

	Run 1		Run 2		Summary	
Summary of Results						
HRM	n	%	n	%	n	%
Number of samples	61	100.0	7	100.0 (11.5)	61	100.0
Analysis passed	50	82.0	7	100.0	57	93.4
WT (total)	32	64.0	6	85.7	38	66.7
WT (skewed HRM curve)	24	75.0	2	33.3	26	68.4
Mutant (total)	18	36.0	1	14.3	19	33.3
Mutant (skewed HRM curve)	0		0		0	
Analysis failed	11	18.0	0			
Pyrosequencing	n	%	n	%	n	%
Number of samples	61	100.0	1	100.0 (1.6)	61	100.0
Analysis passed	60	98.4	1	100.0	61	100.0
WT (total)	41	68.3	0		41	67.2
WT (call: WT)	35	58.3			35	
WT (call: potential low level mutation)	6	10.0	0		6	
Mutant	19	31.7	1	100.0	20	32.8
Analysis failed	1	1.6	0			
Concordance of Results	HRM		Pyrosequencing			
	n	%	n	%		
Number of samples	57	100	57	100		
WT (total)	38	66.7	37	64.9		
WT (call: WT)			33	57.9		
WT (call: potential low level mutation)			4	7.0		
Mutant	19	33.3	20	35.1		
Concordant	56	98.2				
Discordant	1	1.8				
Correctly classified WT	37	97.4				
Incorrectly classified WT	1	2.6				
Correctly classified mutant	19	100				
Incorrectly classified mutant	0	0				

### Concordance of HRM and pyrosequencing results

In order to evaluate the diagnostic validity of HRM analysis as a basic test for *KRAS* mutation detection, we compared the results from this assay to pyrosequencing in the 57 samples that could be successfully analysed by both methods. *KRAS* status was assigned concordantly for 56 samples (98.2 %; kappa = 0.961), while the result for one sample with a low mutant allele frequency of 13.4 % (#3, Table 1) was inconsistent between HRM and pyrosequencing (Tables 1 and 2). Importantly, pyrosequencing indicated the presence of potential low level mutations (mutant allele frequency < 4.0–5.2 %, cf. Table 1) in four samples that were called WT by HRM. Given that this output is generated due to low signal strength for the potential mutation near the technical detection limit of the pyrosequencing method we finally classified these samples as WT. Conversely, all 19 samples that were clearly identified as mutant by HRM were classified identically by pyrosequencing. Therefore, defining pyrosequencing as the reference method, the specificity of HRM for detection of mutant *KRAS* alleles was 100 %. On the other hand, the specificity for the detection of WT alleles was slightly reduced (97.4 %) due to erroneous interpretation of the HRM curve for the one sample mentioned (#3, Table 1) with a mutant allele frequency only slightly above the sensitivity threshold of the method. When we applied a different HRM assay for the detection of NRAS codon 61 mutations on an independent set of 19 CRC samples, we found a 100 % concordance of results with reports from a reference laboratory (Additional file 1: Table S1). Of note, sensitivity of the *NRAS* HRM assay was comparable to the *KRAS* assay and allowed for reliable identification of mutations at a mutant sample fraction of 20 % (Additional file 2: Figure S1). Taken together, these findings indicate that HRM represents a very reliable basic method for *KRAS* mutation testing.

### Two-step *KRAS* mutation testing in routine diagnostics

**Fig. 3:**
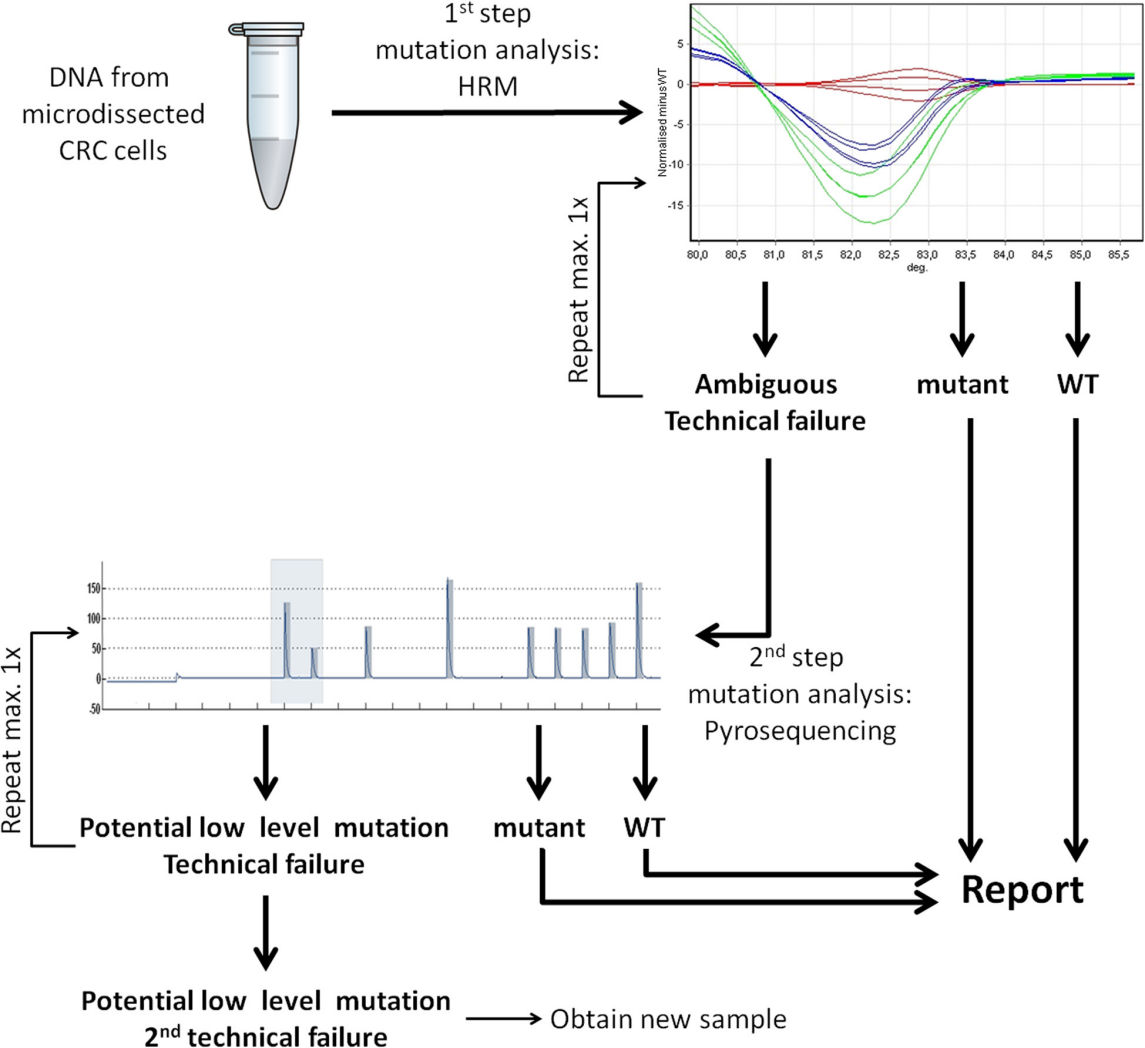
Outline of the two-step procedure for *KRAS* codon 12/13 mutation analysis. Genomic DNA from microdissected colorectal cancer cells from FFPE samples is subjected to HRM of a PCR amplicon spanning the mutation-bearing region of interest. For samples that are clearly identified as *KRAS* mutant or, respectively, WT, the HRM result is incorporated in the final diagnostic report. Samples for which HRM analysis fails technically or which yield ambiguous HRM curves are further evaluated by a second round of HRM and, if results are still invalid, to pyrosequencing. Note that samples for which a WT result is obtained by the diagnostic procedure outlined here require further examination for additional *KRAS* and *NRAS* mutations

To evaluate the actual effectiveness of our two-step analysis platform (Fig. 3) in a routine diagnostic setting, we reviewed effort and outcome of *KRAS* codon 12/13 mutation testing in 120 independent colorectal cancer samples that were examined consecutively in our laboratory according to this concept (Table 3). We found, that *KRAS* status could be determined for 87.5 % of samples by HRM and for 99.2 % of samples in total, when pyrosequencing was applied to samples that could not be successfully analysed by HRM (Table 4). However, for both HRM and pyrosequencing, the failure rate was slightly higher than anticipated based on the observations from our initial 61 sample set (Tables 2 and 4). Also of note, the number of samples that were subjected to pyrosequencing in routine diagnostics exceeded the previously estimated need of this analysis (19/120 = 15.5 % vs. 4/61 = 6.6 %) because 15 samples were directly analysed by pyrosequencing after the first failed HRM run in order to utilize otherwise wasted capacities. Yet, in summary, these data strongly support the rationale of our two-step approach to *KRAS* codon 12/13 mutation analysis, confirming the accuracy of our diagnostic platform.

**Table 3:** Detailed results of *KRAS* codon 12/13 mutation testing in 120 CRC samples

		HRM		Pyrosequencing				
				Run 1		Run 2		
Sample	c_DNA_ [ng/μl]	Run 1	Run 2	Result	% mut. Alleles	Result	% mut. Alleles	Final result
62	541	WT ^a^		WT				WT
63	29	WT ^a^		WT				WT
64	378	WT						WT
65	342	WT ^a^	WT					WT
66	176	failed	WT ^a^	G12V	3.2	G12V	5.2	WT
67	139	mut						mut
68	520	WT						WT
69	42	WT						WT
70	202	WT						WT
71	157	mut						mut
72	259	mut						mut
73	145	WT						WT
74	21	failed	failed	WT				WT
75	22	failed		WT				WT
76	66	mut						mut
77	55	mut						mut
78	199	mut						mut
79	197	WT						WT
80	171	WT						WT
81	55	failed		G12V	41.1			mut
82	231	failed		WT				WT
83	250	failed		WT				WT
84	57	failed	WT ^a^					WT
85	21	mut						mut
86	248	WT						WT
87	258	WT						WT
88	83	failed		failed		failed		N/A
89	38	failed		WT				WT
90	279	WT						WT
91	122	failed		WT				WT
92	129	failed		Low Mut.	5.2	Low Mut.	8.2	mut
93	58	WT						WT
94	129	WT						WT
95	254	WT						WT
96	373	WT						WT
97	158	WT						WT
98	96	mut						mut
99	22	WT						WT
100	30	mut						mut
101	26	mut^a^	mut^a^	G13D	7.3			mut
102	49	WT						WT
103	47	WT						WT
104	43	WT						WT
105	54	WT						WT
106	363	failed	mut					mut
107	521	failed	WT					WT
108	199	mut						mut
109	260	WT						WT
110	67	WT						WT
111	103	WT						WT
112	24	mut						mut
113	150	WT						WT
114	5	WT						WT
115	25	WT						WT
116	33	WT						WT
117	26	mut						mut
118	72	WT						WT
119	16	failed		G12V	12.8			mut
120	33	failed		WT				WT
121	48	WT						WT
122	74	WT						WT
123	474	mut						mut
124	431	mut						mut
125	66	mut						mut
126	143	mut						mut
127	49	failed	WT					WT
128	143	WT						WT
129	122	mut						mut
130	139	WT						WT
131	21	failed		WT				WT
132	39	failed		WT				WT
133	128	failed		G12D	26.7			mut
134	60	mut						mut
135	330	failed	mut					mut
136	165	mut						mut
137	213	mut						mut
138	31	failed	mut					mut
139	156	mut						mut
140	59	WT						WT
141	68	WT						WT
142	164	WT						WT
143	238	mut						mut
144	12	mut						mut
145	33	failed	WT					WT
146	81	WT						WT
147	11	WT						WT
148	13	mut						mut
149	71	WT						WT
150	11	mut						mut
151	40	failed	WT					WT
152	50	WT						WT
153	128	mut						mut
154	146	WT						WT
155	69	WT						WT
156	182	WT						WT
157	32	failed	WT					WT
158	142	WT						WT
159	53	WT						WT
160	91	failed	WT					WT
161	334	WT						WT
162	86	failed	WT					WT
163	61	WT						WT
164	64	WT						WT
165	141	WT						WT
166	271	WT						WT
167	40	WT						WT
168	34	failed	WT					WT
169	29	failed	failed	WT				WT
170	354	WT						WT
171	66	WT						WT
172	43	failed	WT					WT
173	114	WT						WT
174	268	WT						WT
175	107	WT						WT
176	170	mut						mut
177	65	failed	mut					mut
178	31	failed	mut					mut
179	61	WT						WT
180	659	mut						mut
181	34	WT						WT

^a^Skewed HRM curve

**Table 4:** Operational analysis of two-step *KRAS* mutation testing of 120 CRC samples

	Run 1		Run 2		Summary	
HRM	n	%	n	%	n	%
Number of samples	120	100.0	20	100.0 (16.7)	120	100.0
Analysis passed	89	74.2	18	90.0	105	87.5
WT (total)	60	67.4	12	66.7	71	67.6
WT (skewed HRM curve)	3	5.0	2	16.7	4	5.6
Mutant (total)	29	32.6	6	33.3	34	32.4
Mutant (skewed HRM curve)	1	3.4	1	16.7	1	2.9
Analysis failed	31	25.8	2	10.0		
Pyrosequencing	n	%	n	%	n	%
Number of samples	19	100.0 (15.8)	3	100.0 (2.5)	19	100.0
Analysis passed	18	94.7	2	66.7	18	94.7
WT	12	66.7	0	0.0	13	72.2
Potential low level mutation	2	11.1	1	50.0	0	0.0
Mutant	4	22.2	1	50.0	5	27.8
Analysis failed	1	5.3	1	33.3	1	
Combined HRM + Pyrosequencing					n	%
Number of samples					120	100.0
Number of HRM runs					140	116.7
Number of pyrosequencing runs					22	18.3
Analysis passed					119	99.2
WT					81	68.1
Mutant					38	31.9
Analysis failed					1	0.8

### Assay costs

In order to estimate the economic benefits of our two-step approach to *KRAS* mutation testing, we compared analysis costs in our routine setting to a pyrosequencing-only platform. Based on current list prices for reagents and consumables, we estimated the assay costs for HRM analysis and pyrosequencing at approximately € 7.50 and € 100, respectively (Table 5). The costs for the essential technical devices for both methods have not been converted to per-sample costs because operation expenses are highly dependent on sample throughput, including not only the *KRAS* mutation assay but also other applications. Moreover, investments for technical equipment are in the same range for pyrosequencing and HRM. Considering the failure rates of each assay in our set of 120 routine samples (23.6 % for HRM and 9.1 % for pyrosequencing), leading to repeated testing of some samples, our two-step approach allows for net reduction of operational costs of approximately 75 % compared to pyrosequencing alone. Moreover, according to our experience, hands-on time for processing the maximum number of samples for one HRM-run (14 + 4 controls) is only half of the time required to prepare and perform a pyrosequencing run at full capacity (22 + 2 controls) (Table 5). Therefore, our concept to maintain two sequential assays for *KRAS* codon 12/13 mutation testing represents cost- and time-effective approach for routine diagnostics.

**Table 5:** Per-sample costs and hands-on time for HRM and pyrosequencing analyses

	HRM	Pyrosequencing
Costs (Euro)		
Reagents	3.40	90.00
Consumables	2.40	3.50
Controls	1.70	8.50
Total	7.50	102.00
Time (minutes)		
14 samples + 4 controls	60	
22 samples + 2 controls		120

Costs for the controls were estimated based on the maximum number of samples that can be processed in one HRM- or pyrosequencing run, respectively. Costs for HRM controls also include DNA isolation from *KRAS* WT and mutant cell lines. Hands-on time is indicated for full capacity runs

## Discussion

Here we present a two-step approach to *KRAS* codon 12/13 mutation testing for mCRC employing HRM analysis and pyrosequencing using the *Therascreeen KRAS* Pyro Kit. Comparing the performance of the two methods in a panel of 61 samples, we observed a 98.2 % concordance of results with a 100 % specificity of HRM for the detection of mutant alleles. Thus, HRM analysis needs methodically independent confirmation of results by pyrosequencing only in exceptional cases and therefore can serve as a filter assay to exclude clearly WT or mutant samples from the more expensive and more laborious pyrosequencing analysis. Specifically, based on our observations reported here, this approach can reduce throughput of the pyrosequencing assay by >85 %, resulting in a >75 % cost reduction compared to using pyrosequencing only. We emphasize, that our comparison of the two methods in the first place aimed on diagnostic accuracy for sequential application in order to establish a reliable and economized platform for *KRAS* mutation testing. Of note, we reached this goal in spite we were able to detect mutant *KRAS* alleles only at a frequency >12.5 % instead of 5 % as reported in the literature [20, 26].

With respect to technical performance, although we successfully applied HRM to very low input samples, we state a clear advantage for the pyrosequencing assay due to lower input requirements and an apparently relatively high susceptibility of HRM to artifacts. More precisely, previous authors have pointed out, that especially WT HRM curves show a certain degree of variation resulting from poor quality of FFPE-derived template DNA, differing salt- or inhibitor concentrations or unspecific amplification [20, 27], that may complicate correct determination of *KRAS* status. Consistent with this notion, 6 of the 7 samples in our 61-sample validation set that were subjected to a second round of HRM analysis due to poor interpretability of first round results were eventually called WT by this method. Conversely, we did not obtain false positive results by HRM, i.e., none of our samples that had been identified as mutant by HRM was found to be WT according to pyrosequencing. Yet, we state that the mutation frequency of *KRAS* codon 12/13 observed in our study was slightly lower than reported in the literature [9, 10], which may be explained by our homogenous patient population from a single center (Marburg, Germany).

Concerning diagnostic value of results from our sequential *KRAS* mutation analysis procedure, it is important to point out that pyrosequencing results include information on the site, type and frequency of the nucleotide exchange, while HRM only allows for categorical discrimination of WT and mutant tumors. According to current standards, such a dual output is actually sufficient to establish the indication for anti-EGFR treatment, although certain authors have suggested that not all *KRAS* mutations are equal regarding outcome in mCRC patients treated with cetuximab [3]. Consequently, as clinical routine testing at present in principle does not require sequence-based analysis, the more differentiated output of the pyrosequencing assay does not warrant the higher costs for this analysis. Therefore, the two-step procedure for *KRAS* mutation testing presented here represents a reasonable diagnostic approach not only from a technical-practical and economical, but also from a clinical perspective. More specifically, using our diagnostic platform focused on *KRAS* codon 12/13 mutation testing, even small diagnostic laboratories can provide accurate and clinically meaningful results within a short processing time for the most relevant genetic alteration that determines a treatment decision for mCRC patients. Consequently, only a small fraction of patient samples has to be sent to an external reference laboratory for further molecular studies in accordance with the current EMA standards and recommendations by the American Society of Clinical Oncology, which state that Ras mutation testing prior to initiation of treatment with cetuximab and panitumumab has to include analysis of both *KRAS* and *NRAS* exons 2, 3 and 4 (codons 12, 13, 59, 61, 117 and 146). Also of note, besides *KRAS* and *NRAS* mutations, alterations in several other genes such as *BRAF* and *PIK3CA* have been proposed to predict outcome with EGFR antibody treatment [28–30]. Thus, identification of patients eligible for cetuximab or panitumumab treatment in fact requires either a broad panel of single mutation tests or a multiplex approach. Optimized methods for DNA melting analysis of short PCR amplicons have been suggested to allow for comprehensive hot spot mutation testing in a clinical setting as they require only standard qPCR equipment. However, each assay requires careful optimization, implying considerable efforts for a diagnostic laboratory to set up all tests on site [31]. Alternatively, next generation sequencing (NGS) with a targeted resequencing approach appears to be a suitable technology for extensive clinically relevant mutation testing in the future, which has already been evaluated for the molecular diagnostics of colorectal cancer [32, 33]. Given the high frequency of *KRAS* codon 12/13 mutations compared to other *KRAS*- or *NRAS* mutations and the fact that these mutations occur mutually exclusive [10], it still seems reasonable, to filter the samples that actually need advanced testing method as proposed here. Thus, a two-step approach including HRM analysis of *KRAS* codon 12/13 mutations followed by next generation targeted resequencing might be the most attractive implementation for routine *KRAS* mutation diagnostics in the future.

## Conclusion

We present a diagnostically reliable and cost-effective two-step approach to *KRAS* codon 12/13 mutation testing of CRC samples prior to initiation of treatment with anti-EGFR antibodies. The platform appears to be especially attractive for small to medium diagnostic laboratories that don’t have the capacities to maintain an extensive spectrum of rare mutation tests according to regulatory standards for diagnostic laboratories [34] or to adopt NGS-technology with its complex associated infrastructure including bioinformatics.

## Abbreviations

CRC, colorectal cancer; EMA. European medical agency; FDA, U.S. food and drug administration; HRM, high resolution melting analysis; LOD, limit of detection; mCRC, metastatic colorectal cancer; NGS, next generation sequencing; PCR, polymerase chain reaction; qPCR, quantitative polymerase chain reaction; WT, wildtype; UICC, Union internationale contre le cancer.

## Additional files

Additional file 1: Supplementary method and Table S1. HRM analysis of NRAS codon 61 and Results of NRAS codon 61 mutation testing in 19 CRC samples. (DOCX 22 kb) Additional file 2: Figure S1. HRM analysis of NRAS codon 61. (TIF 553 kb)
