# Supplementary material for: A rational two-step approach to KRAS mutation testing in colorectal cancer using high resolution melting analysis and pyrosequencing
Source: BMC Cancer. 2016 Aug 2;16:585. doi: 10.1186/s12885-016-2589-2 (PMC4971616; doi:10.1186/s12885-016-2589-2)
Supplement: Additional file 1: — Supplementary method and Table S1. HRM analysis of NRAS codon 61 and Results of NRAS codon 61 mutation testing in 19 CRC samples. (DOCX 22 kb) [file 12885_2016_2589_MOESM1_ESM.docx]

**Supplementary Information**

**Supplementary Method**

**High resolution melting analysis of NRAS codon 61**

HRM analysis for *NRAS* codon 61 mutations was performed essentially as described for *KRAS* codon 12/3 using the primers *NRAS*-codon 61_F 5´-cacacccccaggattcttac*-*3´and *NRAS*-codon 61_R 5´-tggcaaatacacagaggaagc-3´, and the following temperature conditions: one cycle 95°C/2min, 40 cycles 95°C/15sec – 60.6°C/15sec - 72°C/15sec, one cycle 95°C/1sec, pre-melt conditioning at 72°C/90sec, HRM-ramp from 72°C to 95°C rising at 0.2°C per step/wait 2sec each step. Controls in each HRM run included a no-template-control, a WT control (gDNA from healthy donor) and three mutation controls (gDNA from the cell line HL-60, *NRAS* codon 61 CAA→CTA/heterozygous corresponding to Q61L and two plasmids: NRAS_61-CAC, corresponding to Q61H, and NRAS_61-CGA, corresponding to Q61R). Plasmids containing mutant *NRAS* codon 61 sequences were constructed by site directed mutagenesis from a WT gDNA template as confirmed by sequencing using the QuikChange XL Site-Directed Mutagenesis Kit (Stratagene) according to the manufacturer´s instructions with the primers NRAS_61-CAC_F 5´-ctggatacagctggacacgaagagtacagtgccatgag-3´ and NRAS_61-CAC_R 5´-ctcatggcactgtactcttcgtgtccagctgtatccag-3´, or, respectively, NRAS_61-CAA_F 5´- ctggatacagctggacgagaagagtacagtgccatgag-3´ and NRAS_61-CAA_R 5´-ctcatggcactgtactcttctcgtccagctgtatccag-3´. PCR products were cloned into pCR II-TOPO vector using the TOPO TA Cloning Kit (Invitrogen) as recommended and mutations were verified by sequencing. To determine the sensitivity of the *NRAS* codon 61 HRM assay, DNA from a CRC sample with a confirmed Q61L mutation was diluted in WT DNA and HRM curves for each dilution step were compared to HL-60.

**Supplementary Figure**

**Fig. S1: HRM analysis of NRAS codon 61**

A CRC sample was analysed for NRAS codon 61 status by HRM and compared to the positive controls, HL-60 (CAA→CTA/heterozygous) and two plasmids (NRAS_61-CAC: CAA→CAC and NRAS_61-CGA: CAA→CAC). Normalised fluorescence and difference graphs are indicated. The CRC sample carries the same mutation as HL-60 (top panel). The experiment was repeated for a dilution series of this sample in WT genomic DNA: Mutant amplicons could be clearly distinguished for a sample fraction of 20%, corresponding to a mutant allele frequency of 10% if the mutation in this sample is heterozygous (zygosity unknown) (middle and bottom panel).

**Supplementary Table**

**Tab. S1: Results of NRAS codon 61 mutation testing in 19 CRC samples**

|  | **KRAS codon 12/13** | **NRAS codon 61** | |
| --- | --- | --- | --- |
| **Sample** |  | **HRM** | **Reference** |
| **182** | WT | WT | WT |
| **183** | WT | WT | WT |
| **184** | WT | WT | WT |
| **185** | WT | WT | WT |
| **186** | WT | WT | WT |
| **187** | WT | WT | WT |
| **188** | WT | WT | WT |
| **189** | WT | WT | WT |
| **190** | WT | WT | WT |
| **191** | WT | **mut** | **mut** |
| **192** | WT | WT | WT |
| **193** | WT | WT | WT |
| **194** | WT | WT | WT |
| **195** | WT | WT | WT |
| **196** | WT | WT | WT |
| **197** | WT | WT | WT |
| **198** | WT | **mut** | **mut** |
| **199** | WT | WT | WT |
| **200** | WT | WT | WT |
