# Supplementary figures and images for: A rational two-step approach to KRAS mutation testing in colorectal cancer using high resolution melting analysis and pyrosequencing
Source: BMC Cancer. 2016 Aug 2;16:585. doi: 10.1186/s12885-016-2589-2 (PMC4971616; doi:10.1186/s12885-016-2589-2)

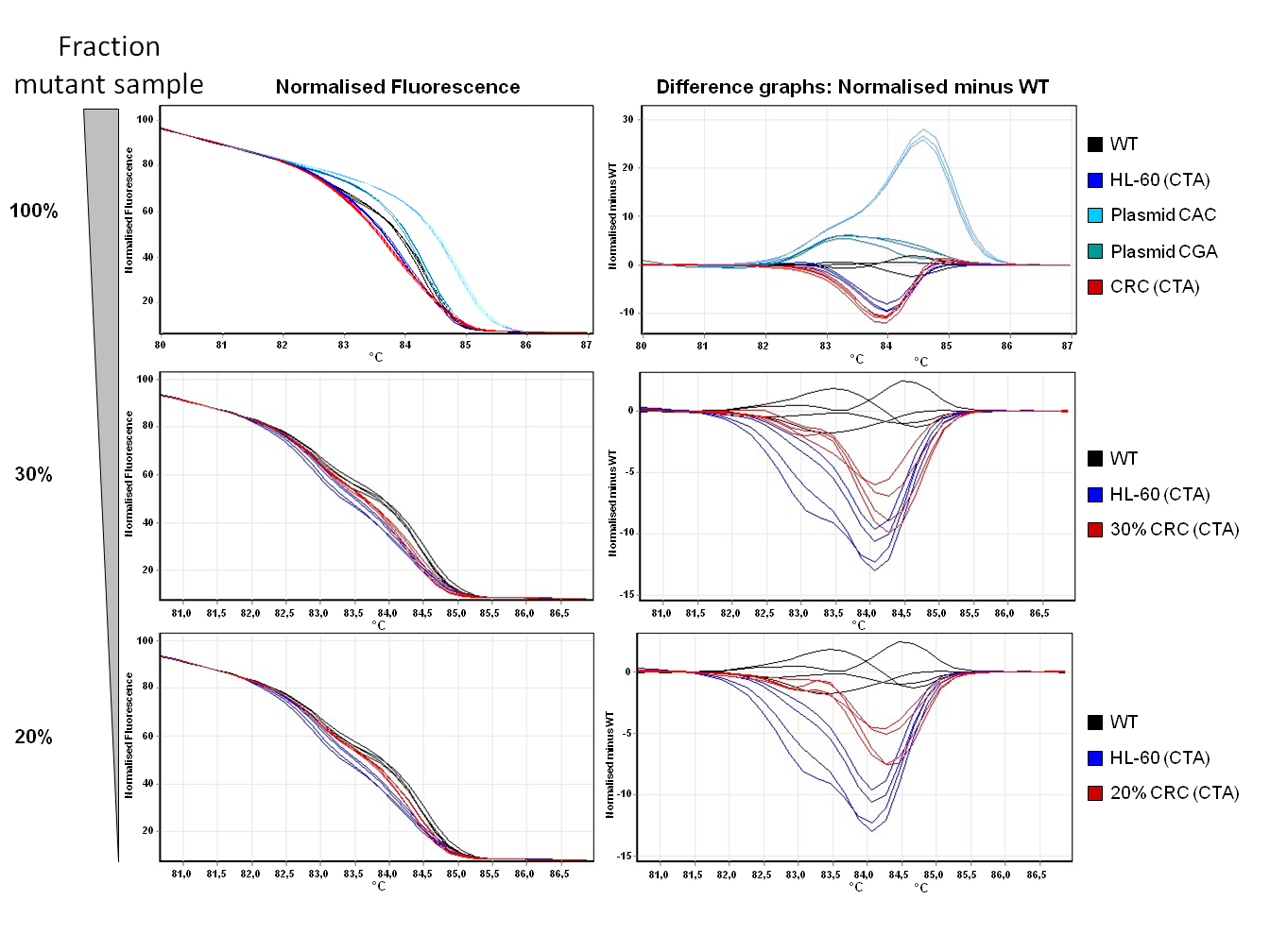

Supplement: Additional file 2: Figure S1. — HRM analysis of NRAS codon 61. (TIF 553 kb) [file 12885_2016_2589_MOESM2_ESM.tif]
